# PReS-FINAL-2335: Preliminary analysis of 85 patients with mevalonate kinase deficiency from the eurofever registry

**DOI:** 10.1186/1546-0096-11-S2-P325

**Published:** 2013-12-05

**Authors:** N Ter Haar, H Lachmann, P Woo, A Simon, A Meini, P Dolezalova, C Modesto, S Stojanov, B Bader-Meunier, A Insalaco, E Hoppenreijs, E Gallo, N Ruperto, J Frenkel, M Gattorno

**Affiliations:** 1 Paediatric Rheumatology International Trials Organisation (PRINTO), Eurofever Project, Genoa, Italy

## Introduction

Mevalonate kinase deficiency (MKD) is a rare autoinflammatory disease, caused by mutations in the isoprenoid pathway that lead to fever episodes. Approximately 300 MKD patients are known.

## Objectives

Our objective is to describe the clinical features and therapeutic response in a large group of MKD patients, in order to increase knowledge of this rare disease.

## Methods

The Eurofever registry (EAHC Project No. 2007332) is retrospectively collecting clinical information on anonymized patients affected by autoinflammatory diseases. Cases were independently validated by an MKD expert.

## Results

Eighty-two patients harbouring two *MVK *mutations and three patients with one *MVK *mutation in combination with elevated urinary excretion of mevalonic acid were used for analysis. The group consisted of 39 males and 46 females. Median age at onset was 6 months; in 60 patients (70.6%) the disease started within the first year. Median disease duration at enrolment in the registry was 13.1 years. The majority of patients were Caucasian. The most frequent mutation was V377I, occurring in 70 patients, including 12 patients that were homozygous for this mutation. I268T was the second most frequent mutation (n = 23). The majority of patients (75 of 85) had a recurrent disease course. The median duration of a fever episode was 5 days, 80.8% had a duration of 3 to 7 days. Median attack frequency was 12 per year. Triggers for disease activation were known in 38 patients and included vaccination (n = 28), stress (n = 20) and infection (n = 12). Nearly all patients (83 of 85) suffered from gastrointestinal symptoms, mainly diarrhea (n = 66), abdominal pain (n = 65) and vomiting (n = 51). Seventy-one patients had cervical lymphadenopathy. Mucocutaneous symptoms included aphthous stomatitis (n = 47), maculopapular rash (n = 31) and pharyngitis (n = 34). Arthralgia (n = 59) and myalgia (n = 47) were more prominent than arthritis (n = 27). Many patients display malaise (n = 47), fatigue (n = 50) and weight loss (n = 44), sometimes independent of fever attacks. IgD-levels were increased in 42 of 61 tested patients and urinary mevalonic acid excretion was increased in 27 of 28 tested patients. Non-steroidal anti-inflammatory drugs (NSAIDs) were mainly given during attacks and relieved symptoms in 33 of 46 patients, including 5 patients who noted complete suppression of inflammatory symptoms. A complete response on corticosteroids was seen in 13 of 48 patients (all used during attacks), another 29 patients reported improvement. Anakinra was completely effective in 5 of 26 patients and partially effective in 17 others. Etanercept was used by 19 patients, one patient reported complete response, 11 patients some improvement; failure was noted in 7 patients. Statins failed or worsened disease in 7 patients, 4 patients reported some improvement.

## Conclusion

This study describes the clinical characteristics and response to therapy in a large international cohort of MKD patients.

## Disclosure of interest

None declared.

